# Prevalence of metabolic syndrome among ethnic groups in China

**DOI:** 10.1186/s12889-020-8393-6

**Published:** 2020-03-06

**Authors:** Xuzhen Qin, Ling Qiu, Guodong Tang, Man-Fung Tsoi, Tao Xu, Lin Zhang, Zhihong Qi, Guangjin Zhu, Bernard M. Y. Cheung

**Affiliations:** 1 Department of Laboratory Medicine, Chinese Academy of Medical Sciences & Peking Union Medical College Hospital, Beijing, 100730 China; 2 Department of Cardiology, Beijing Hospital of Health Ministry, Beijing, 100730 China; 3 Department of Medicine, University of Hong Kong, Queen Mary Hospital, 102 Pokfulam Road, Hong Kong, China; 4 Department of Epidemiology and Statistics, Institute of Basic Medical Sciences, Chinese Academy of Medical Sciences & School of Basic Medicine, Peking Union Medical College, Beijing, 100005 China; 5 Department of Pathophysiology, Institute of Basic Medical Sciences, Chinese Academy of Medical Sciences & School of Basic Medicine, Peking Union Medical College, Beijing, 100005 China

**Keywords:** Metabolic syndrome, Ethnic group, China

## Abstract

**Background:**

Metabolic syndrome (MetS) is common in China, which has a multi-ethnic population of 1·3 billion. We set out to determine the prevalence of MetS and its components in different ethnic groups.

**Methods:**

This nationwide cross-sectional survey involved 24,796 participants from eight ethnicities in six provinces in China from 2008 to 2011. MetS was defined using the modified National Cholesterol Education Program Adult Treatment Panel III criteria. Results were analysed using SPSS version 22·0 in 2018. Logistic regression was used for deriving odds ratios and 95% confidence intervals of risk factors for the MetS.

**Results:**

The prevalence of MetS increased with age from 3·60% to 21·68%. After age standardization, the prevalence of MetS, in descending order, was 35·42% (Korean), 22·82% (Hui), 19·80% (Han), 13·72% (Miao), 12·90% (Tujia), 12·04% (Li), 11·61% (Mongolian), 6·17% (Tibetan). Korean ethnicity was associated with a higher prevalence in five components of MetS, while Tibetan ethnicity was associated with lower prevalence except decreased HDL cholesterol. Logistic regression analyses showed that age, drinking and being non-Tibetan were associated with a higher risk of MetS.

**Conclusions:**

Within one country, albeit a large one, the prevalence of MetS can vary greatly. Chinese of Korean ethnicity had a much higher prevalence than Tibetan ethnicity. Measures to tackle MetS should be tailored to the ethnic groups within a population.

## Background

Metabolic Syndrome (MetS) is a cluster of related abnormalities that include abdominal obesity, insulin resistance, dyslipidaemia and elevated blood pressure [1, 2]. The National Cholesterol Education Program’s Adult Treatment Panel III (NCEP: ATPIII), World Health Organization (WHO) and International Diabetes Federation (IDF) use this syndrome to highlight the risk of patients developing cardiovascular disease (CVD) and type 2 diabetes (T2DM) [1, 3]. We previously reported that the prevalence of MetS in the United States was 33·6% in the adult population [4]. MetS predicts the development of diabetes [5] and hypertension [6], and is also associated with coronary artery disease (CAD) [7] and increased mortality [8].

While the natural history of MetS and how it develops have been well described in Hong Kong Chinese, a large number of observational studies have also been conducted in China, the population of which is mostly of Han ethnicity [9, 10]. A meta-analysis showed that the pooled prevalence of MetS in China was 24·5% among subjects aged over 15 years. Individuals living in urban areas had a higher risk of having MetS than those living in rural areas [11]. However, China is a multi-ethnic country with at least 55 ethnic groups. There are few studies focused on MetS among different ethnic groups in China, and most of these studies focused on a single ethnic group [7, 12, 13]. The heterogeneity in study design, inclusion criteria and definition of MetS makes it difficult to compare the prevalence in different ethnic groups.

Building on the Expansion Investigation of Human Physiology Constant Study in China [14], we made use of unified inclusion criteria and centralized measurements to determine the prevalence of Metabolic Syndrome (MetS) among different ethnicities in six provinces of China using the modified NCEP: ATPIII criteria [15].

## Methods

### Description of the study

The Expansion Investigation of Human Physiology Constant was a nationwide cross-sectional, random and multistage clustering sampled survey in China that is part of the National Constitution and Health Database in 2008–2011 [16]. Our previous studies based on the National Constitution and Health Database had shown several factors associated with cardiovascular disease [14, 17]. This study was expanded to six provinces and eight ethnic groups according to the geographical, ethnical and economic characteristics from the previous studies. All study personnel was trained with standardised working manuals and surveys before they conducted this study. Sample processing, testing and quality control were conducted by certified personnel in a central laboratory (Department of Laboratory Medicine in Peking Union Medical College Hospital) to ensure the consistency and stability of the measurements.

### Subjects

**Fig. 1:**
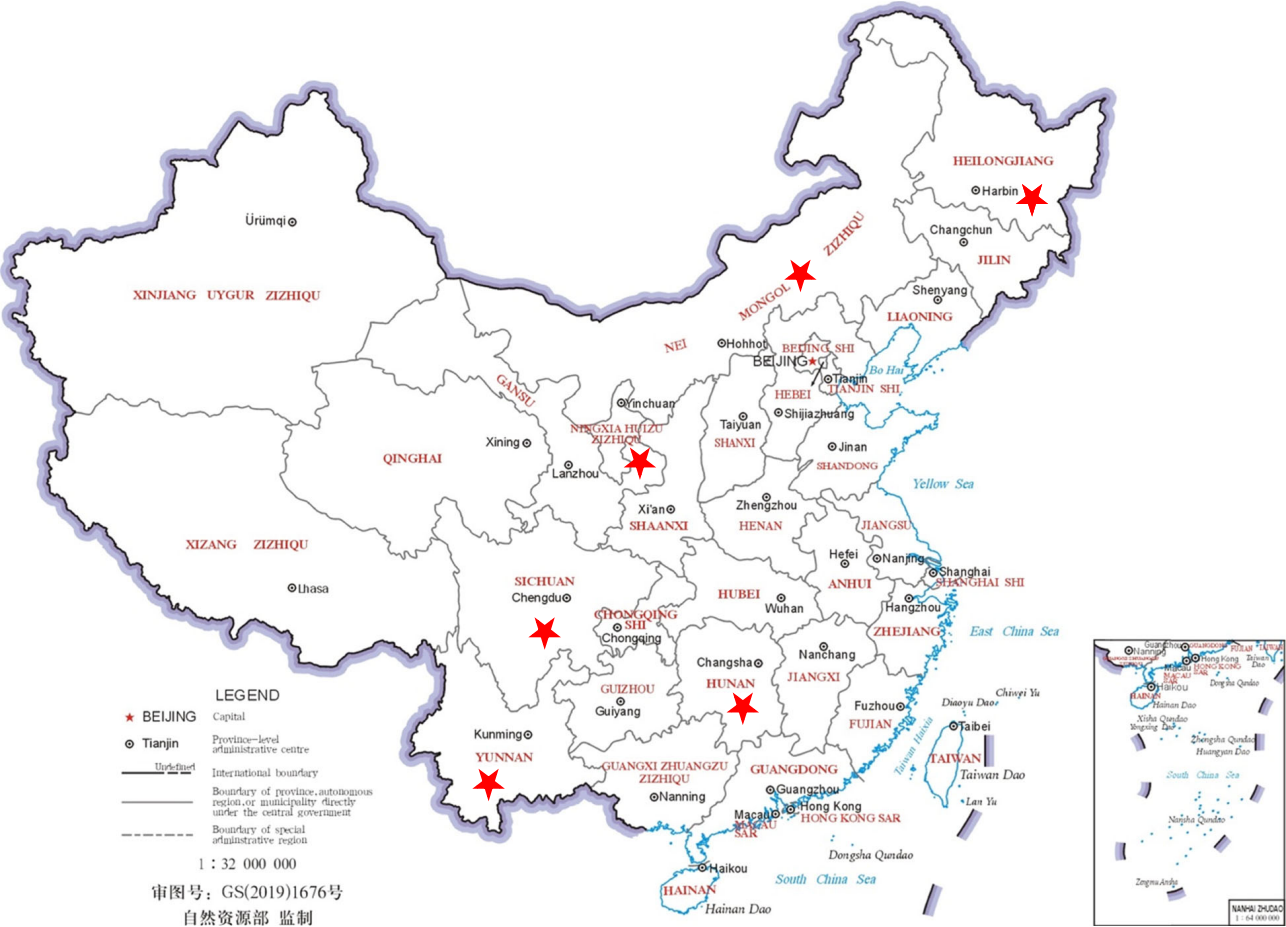
Distributions of participants in China

Participants aged 8–86 years were recruited from six provinces (Inner Mongolian, Heilongjiang, Ningxia, Hunan, Yunnan, Sichuan) using a random, multistage cluster-sampling method as shown in Fig. 1. Eight ethnic groups, including Han, Mongolian, Korean, Hui, Miao, Li, Tibetan and Tujia, were enrolled in this study. Inclusion criteria were absence of self-reported systemic diseases, such as CAD, renal disease, autoimmune disease, hypersensitivity, gastrointestinal disease, pulmonary disease, and cancer. Considering different customs of ethnic groups, we included participants aged 8–86. Exclusion criteria included significant abnormalities on physical examination, and having a fever, acute illness, or hospitalization within 15 days. This study was approved by the Ethics Committee of Institute of Basic Medical Sciences Chinese Academy of Medical Sciences (No·005–2008). Written consents were obtained from all participants.

### Data collection

Participants were required to complete a demographic questionnaire and a health behaviour questionnaire, which included smoking, alcohol consumption and physical activity. Trained personnel assisted the participants in completing the questionnaires.

### Measurements

All participants were asked to avoid smoking and heavy physical activity for at least 2 h before the physical examinations, which included resting blood pressure, height, weight, and waist circumference (WC). Two blood pressure measurements were taken using OMRON HEM-7000 electronic sphygmomanometer (OMRON HealthCare, Kyoto, Japan) after the participants had rested in a sitting position for at least 5 min.

Participants were required to fast for 8 to 12 h before blood sampling. Fasting blood glucose (GLU), total cholesterol, triglycerides, high-density lipoprotein cholesterol (HDL cholesterol), and low-density lipoprotein cholesterol (LDL cholesterol) were analysed using a Hitachi 7020 chemistry analyser (Hitachi, Tokyo, Japan). Body mass index (BMI) was a person’s weight in kilograms divided by the square of height in meters.

### Definition of metabolic syndrome

The modified ATP III criteria were applied in the diagnosis of MetS, which requires the presence of at least three out of five factors [15]: (i) Abdominal density as defined by waist circumference ≥ 90 cm in men and ≥ 80 cm in women; (ii) triglycerides ≥1·7 mmol/L; (iii) HDL cholesterol *<* 1·03 mmol/L in men and *<* 1·29 mmol/L in women; (iv) systolic blood pressure (SBP) ≥130 mmHg or diastolic blood pressure (DBP) ≥ 85 mmHg; (v) GLU ≥5·6 mmol/L as impaired fasting glucose (IFG).

### Statistical analysis

Results were analysed using SPSS version 22·0 (IBM SPSS Statistics, Armonk, NY, USA) in 2018. Descriptive statistics were expressed as frequency (percentage) for categorical data or mean ± SD or median (interquartile range) for continuous variables. Missing data were less than 3% for all included variables. Continuous variables were compared among groups using one-way ANOVA and Kruskal-Wallis tests as appropriate. Categorical variables were compared among groups using Chi-square test. Logistic regression was used to identify the predictors for MetS. Gender, age, ethnicities, exercises, smoking status, drinking status were included in the model. Age groups and gender were entered into the first adjusted model. Other predictor variables were entered stepwise if *P* < 0·05 and removed if *P* > 0·10. Exercise was added as a covariate in the second adjusted model and ethnicities, drinking status, smoking status were applied in the third adjusted model. Odds ratios (ORs) and 95% confidential interval (95% CI) were estimated. A two-tailed *P* < 0·05 was considered statistically significant. Data from the Sixth National Population Census of the People’s Republic of China provided by the National Statistics Bureau of China were used as the standard population. Age-specific prevalence and age-standardized prevalence were estimated from the standard population.

## Results

**Fig. 2:**
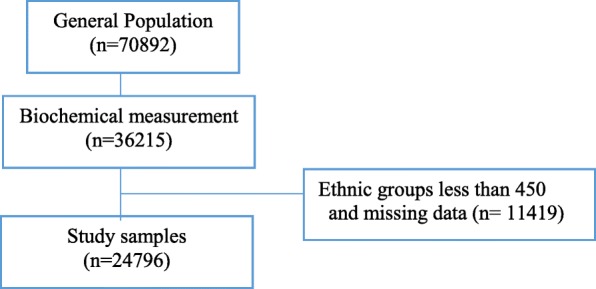
A flow diagram for inclusion of study samples

A total of 24,796 participants were enrolled in this study (Fig. 2). The characteristics are summarized in Supplementary Table 1. There was a significant difference in the median age among participants from different provinces and ethnic groups (shown in Supplementary Table 1, 2 and 3). Compared with Hans in the same province, participants from Tibetan, Miao, Tujia, Mongolian, Li ethnic groups were younger (*P* < 0·001).

### The prevalence of MetS in eight ethnic groups of China

**Fig. 3:**
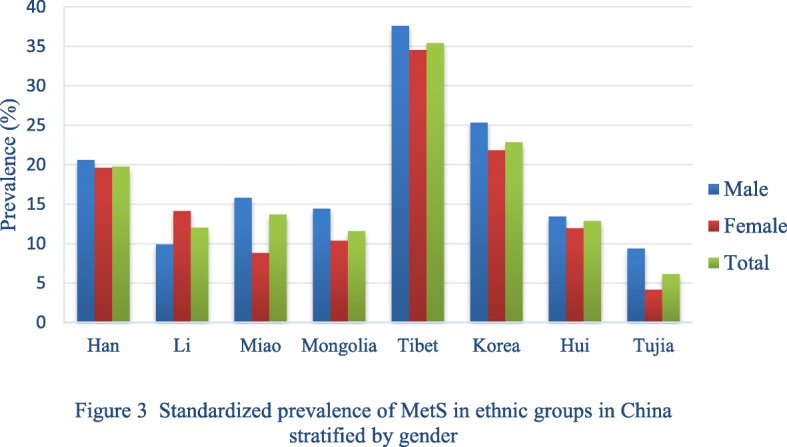
Standardized Prevalence of MetS in ethnic groups in China stratified by gender

The age, gender and ethnic-specific crude and standardised prevalence of MetS are summarised in Table 1. The prevalence of MetS in males (13·5%) was slightly higher than in females (12·6%) (*P* = 0·28). The prevalence of MetS was substantially higher above age 25 (24·7%) compared with age ≤ 25 (2·3%) (*P* < 0·001). There was a significant difference in MetS prevalence among ethnic groups (*P* < 0·001). Korean Chinese had the highest prevalence of 35·42, followed by Hui, Han, Miao, Tujia, Li, Mongolian and Tibetan (Table 1, Fig. 3).

**Table 1:** The prevalence of MetS stratified by age, gender and eight ethnic groups in China

Ethnic groups	Gender	Age group						Crude prevalence (%)	Standardised prevalence (%)
		8–12(%)	13–17(%)	18–24(%)	25–39(%)	40–59(%)	> 60(%)		
Han	M(%)	0.90(0.31–1.48)	2.64(1.91–3.37)	3.50(2.38–4.62)	24.01(21.70–26.32)	30.22(27.98–32.47)	26.81(23.77–29.86)	14.60(13.81–15.40)	20.59(20.2–21.0)
	F(%)	1.74(0.92–2.56)	2.73(2.01–3.45)	1.31(0.57–2.05)	9.85(8.35–11.35)	29.28(27.26–31.30)	46.93(43.19–50.67)	14.02(13.26–14.78)	19.63(19.2–20.1)
	Total(%)	1.31(0.81–1.82)	2.69(2.17–3.20)	2.47(1.78–3.16)	16.40(15.04–17.77)	29.71(28.21–31.21)	36.01(33.57–38.44)	14.30(13.75–14.85)	19.80(19.5–20.1)
Li	M(%)	1.18(0.03–6.38)	2.80(0.76–4.84)	3.23(0.67–9.14)	11.96(8.13–15.78)	16.38(11.62–21.14)	16.00(1.63–30.37)	8.95(7.14–10.75)	9.94(9.0–10.9)
	F(%)	5.80(0.28–11.31)	4.39(1.73–7.04)	2.70(0.33–9.42)	13.21(9.25–17.18)	21.25(16.39–26.10)	33.33(15.55–51.11)	12.62(10.51–14.73)	14.15(13.1–15.2)
	Total(%)	3.25(0.45–6.05)	3.56(1.90–5.22)	2.99(0.41–5.58)	12.59(9.83–15.35)	19.01(15.59–22.43)	25.00(13.23–36.77)	10.77(9.38–12.16)	12.04(11.3–12.7)
Miao	M(%)	0.00	0.00	12.50(0.32–52.65)	22.45(10.77–34.13)	22.22(11.96–32.49)	18.52(3.87–33.17)	12.06(8.08–16.04)	15.83(13.7–18.0)
	F(%)	0.00	0.00	10.00(0.25–44.50)	3.85(0.10–19.64)	24.53(12.94–36.11)	0.00	7.69(3.95–11.43)	8.84(6.9–10.8)
	Total(%)	0.00	0.00	11.11(1.38–34.71)	16.00(7.70–24.30)	23.28(15.59–30.97)	14.29(2.69–25.88)	10.18(7.39–12.96)	13.72(12.2–15.3)
Mongolian	M(%)	0.45(0.01–2.50)	2.25(0.07–4.42)	1.52(0.04–8.16)	17.72(9.30–26.14)	22.99(14.15–31.83)	20.83(4.59–37.08)	6.87(4.93–8.81)	14.46(13.2–15.8)
	F(%)	2.75(0.58–4.92)	1.83(0.05–3.62)	3.64(0.14–7.13)	6.67(2.86–10.47)	14.38(8.94–19.81)	36.11(20.42–51.80)	6.73(5.10–8.36)	10.40(9.4–11.4)
	Total(%)	1.59(0.42–2.77)	2.02(0.63–3.41)	2.84(0.39–5.30)	10.25(6.44–14.05)	17.41(12.68–22.14)	30.00(18.40–41.60)	6.79(5.54–8.03)	11.61(10.8–12.4)
Korean	M(%)	4.76(0.99–13.29)	4.28(1.38–7.18)	6.90(0.85–22.77)	42.86(29.90–55.82)	44.83(34.38–55.28)	46.99(36.25–57.73)	22.77(19.11–26.43)	37.60(35.6–39.6)
	F(%)	3.80(0.79–10.70)	6.07(3.42–8.72)	4.55(0.12–22.84)	16.46(8.28–24.63)	45.83(35.87–55.80)	61.90(52.62–71.19)	20.89(17.87–23.92)	34.52(32.8–36.2)
	Total(%)	4.23(0.92–7.53)	5.40(3.42–7.38)	5.88(1.23–16.24)	27.41(19.88–34.93)	45.36(38.14–52.57)	55.32(48.21–62.43)	21.68(19.35–24.02)	35.42(34.1–36.7)
Hui	M(%)	2.00(0.24–7.04)	1.08(0.22–3.12)	5.99(2.39–9.59)	35.51(26.45–44.58)	36.60(29.82–43.38)	34.15(19.63–48.66)	15.56(13.17–17.94)	25.32(23.9–26.7)
	F(%)	1.63(0.20–5.75)	1.71(0.22–3.19)	0.98(0.12–3.50)	12.39(6.31–18.46)	30.30(23.29–37.32)	75.00(57.68–92.32)	9.87(7.94–11.80)	21.83(20.5–23.1)
	Total(%)	1.79(0.05–3.54)	1.40(0.44–2.37)	3.23(1.43–5.03)	23.64(18.02–29.25)	33.70(28.81–38.59)	49.23(37.08–61.38)	12.66(11.13–14.19)	22.82(21.9–23.8)
Tujia	M(%)	0.00	0.00	4.76(0.58–16.16)	11.69(4.51–18.86)	26.00(17.40–34.60)	15.63(3.04–28.21)	12.61(9.05–16.18)	13.45(11.7–15.2)
	F(%)	0.00	0.00	3.70(0.09–18.97)	1.45(0.04–7.81)	18.75(7.71–29.79)	39.13(19.19–59.08)	8.40(4.88–11.93)	11.99(10.0–14.0)
	Total(%)	0.00	0.00	4.35(0.91–12.18)	6.85(2.75–10.95)	23.65(16.80–30.49)	25.45(13.94–36.97)	10.86(8.31–13.41)	12.90(11.6–14.2)
Tibetan	M(%)	1.04(0.03–5.67)	0.39(0.01–2.17)	1.69(0.04–9.09)	11.88(5.57–18.19)	18.75(7.71–29.79)	11.11(2.35–29.16)	4.62(2.92–6.32)	9.38(8.2–10.5)
	F(%)	0.00	1.67(0.22–3.12)	1.32(0.03–7.11)	5.10(1.66–8.54)	7.22(2.07–12.37)	5.88(0.15–28.69)	2.82(1.66–3.98)	4.20(3.5–4.9)
	Total(%)	0.44(0.01–2.41)	1.08(0.22–1.94)	1.48(0.18–5.25)	7.75(4.49–11.01)	11.03(5.93–16.13)	9.09(0.60–17.59)	3.59(2.60–4.58)	6.17(5.6–6.8)

Data is expressed as prevalence (95% confidence interval)

### The prevalence of MetS components

**Fig. 4:**
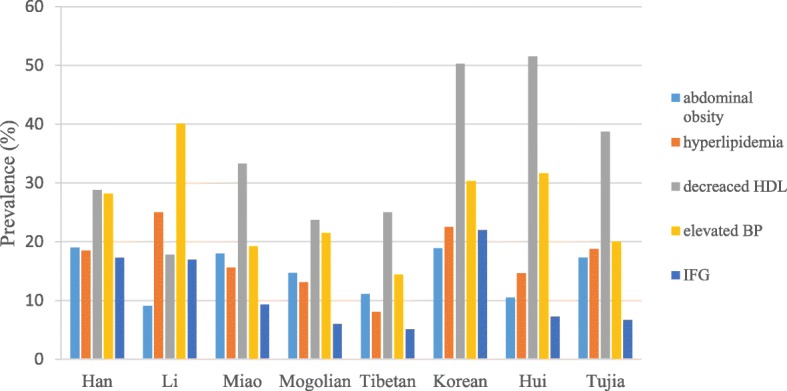
The prevalence of MetS components in ethnic groups in China

Figure 4 shows the prevalence of components of MetS in different ethnic groups. The highest prevalence of abdominal obesity, hyperlipidaemia, decreased HDL cholesterol, elevated blood pressure and IFG were found in Han, Li, Hui, Li and Korean ethnicities, respectively; while the lowest prevalence of the components of MetS appeared in Li and Tibetan ethnicities (Fig. 4). In all ethnic groups, the prevalence of decreased HDL cholesterol and elevated blood pressure were the two highest among the five components, while IFG was less prevalent, especially in Miao, Mongolian, Tibetan, Hui and Tujia ethnicities.

### Predictors for MetS

Predictors for MetS are summarized in Table 2. Age, gender, smoking, alcohol consumption and ethnic groups were all significant risk factors for MetS in the crude model (*p* < 0·001, *p* = 0·027, *p* < 0·001, *p* < 0·001, *p* < 0·001, respectively). Compared to females, males had an increased risk of MetS [OR (95% CI): 1·087 (1·009–1·171), *p* = 0·027]. However, maleness [1·028 (0·930–1·137), *p* = 0·059] and smoking [0·908 (0·806–1·022), *p* = 0·110] were not significant factors for MetS after multivariable adjustment. Compared to participants aged 8 to 12 years, the OR increased with age, and was highest in participants aged 60 to 86 years [34·117 (24·673–47·177), *p* < 0·001]. Relative to Tibetans, all other ethnicities were associated with increased risk of MetS. Korean Chinese had the highest adjusted risk for MetS [5·989 (4·249–8·442), *p* < 0.001], followed by Hui [4·020 (2·859–5·653), *p* < 0.001], Han [2·975 (2·194–4·034), *p* < 0.001], Li [2·096 (1·487–2·954), *p* < 0·001], Miao [1·961 (1·262–3·048), *p* = 0·003], Mongolian [1·835 (1·272–2·648), *p* = 0·001] and Tujia [1·753 (1·166–2·637), *p* = 0·007] ethnicities. MetS was not significantly associated with exercise in both crude and adjusted models (*P* = 0·140, *P* = 0·710, *P* = 0·710, *P* = 0·905, respectively).

**Table 2:** Predictors of metabolic syndrome

Parameters		Crude model	Adjusted model 1	Adjusted model 2	Adjusted model 3
Male		1·087 (1·009–1·171)	1·110 (1·024–1·202)	1·110 (1·025–1·203)	1·028 (0·930–1·137)
Exercise		0·941 (0·867–1·020)	1·017 (0·931–1·110)	1·017 (0·931–1·110)	1·006 (0·911–1·111)
Smoking Status		1·867 (1·702–2·048) ^	0·977 (0·873–1·093)	0·979 (0·875–1·096)	0·908 (0·806–1·022)
Drinking Status		2·159 (1·975–2·360) ^	1·322 (1·186–1·472) ^	1·320 (1·184–1·470) ^	1·348 (1·203–1·509) ^
Age groups	8–12	1 (reference)	1 (reference)	1 (reference)	1(reference)
	13–17	1·767 (1·282–2·435)	1·770 (1·285–2·440) ^	1·769 (1·283–2·438) ^	1·712 (1·208–2·425)
	18–24	1·870 (1·306–2·677)	1·867 (1·304–2·674)	1·863 (1·301–2·669)	1·727 (1·176–2·535)
	25–39	12·191 (9·090–16·350) ^	12·242 (9·128–16·418) ^	12·238 (9·125–16·414) ^	11·67 (8·452–16·113) ^
	40–59	26·041 (19·516–34·747) ^	26·16 (19·605–34·907) ^	26·162 (19·606–34·910) ^	24·583 (17·892–33·776) ^
	60–86	38·350 (28·512–51·584) ^	38·225 (28·418–51·416) ^	38·176 (28·38–51·354) ^	34·117 (24·673–47·177) ^
Ethnic groups	Tibetan	1 (reference)	1 (reference)	1 (reference)	1(reference)
	Han	4·482 (3·358–5·982) ^	2·917 (2·165–3·930) ^	2·916 (2·165–3·929) ^	2·975 (2·194–4·034) ^
	Li	3·243 (2·356–4·465) ^	1·991 (1·431–2·771) ^	1·984 (1·417–2·778) ^	2·096 (1·487–2·954) ^
	Miao	3·043 (2·004–4·620) ^	1·877 (1·213–2·903)	1·877 (1·214–2·904)	1·961 (1·262–3·048)
	Mongolian	1·955 (1·382–2·765) ^	1·815 (1·266–2·602)	1·815 (1·266–2·602)	1·835 (1·272–2·648)
	Korean	7·436 (5·419–10·205) ^	6·116 (4·377–8·547) ^	6·112 (4·373–8·543) ^	5·989 (4·249–8·442) ^
	Hui	3·893 (2·835–5·345) ^	3·629 (2·605–5·054) ^	3·625 (2·602–5·052) ^	4·020 (2·859–5·653) ^
	Tujia	3·271 (2·219–4·824) ^	1·721 (1·151–2·575)	1·722 (1·151–2·576)	1·753 (1·166–2·637)

Data is expressed as odds ratio (95% confidence interval)

^*p* < 0·001

Adjusted model 1: adjusted for gender, age groups

Adjusted model 2: adjusted for gender, age groups and exercises

Adjusted model 3: adjusted for gender, age groups, exercises, ethnicities, smoking status and drinking status

## Discussion

This study is the first large-scale multi-ethnic investigation of the prevalence of MetS in China. With 24,796 participants, it had sufficient power to estimate and compare prevalence in subgroups. Such comparisons are also valid and reliable because we had standardised protocols, rigorous quality control and a central clinical laboratory for measurements. The large sample size, standardised measurements and the quality control allowed us to have accurate estimates of the prevalence of MetS in the general population in China. It is a strength of the study to recruit from the age of 8 to 86, which is an uncommonly wide range for an epidemiological study. The study did not recruit subjects below the age of 8 or above the age of 86 for practical reasons.

The prevalence of MetS in Hans in the present study was higher than a previous national study in China [18], but lower or similar to the prevalence in US and Europe [19, 20]. Due to economic development, consumption of dairy products and fast foods had doubled [21, 22], which might have contributed to the increasing trend in MetS. The prevalence of MetS was slightly higher in men in this study, in contrast to some other populations [23].

The prevalence of MetS in Korean, Hui and Mongolian Chinese in our study is relatively higher than previous studies’ in these ethnicities [24–26]. A study using the Korean National Health and Nutrition Examination Survey (KNHANES) data from 2008 to 2013 reported that the prevalence of MetS among the Korean participants aged ≥20 years was 28·9% according to the modified NCEP: ATPIII definition [25]. A study conducted among rural adults in Ningxia in 2008 reported that the age-adjusted prevalence of MetS was 13·7% with International Diabetes Federation definition [26]. In Mongolians aged over 18 years, the prevalence of MetS in men was higher than in women (36·7% vs. 17·8%) [24]. However, a study of Tibetan immigrants in India aged over 20 years reported a prevalence of 10·6% in men and 33·3% in women [27]. In our study, Tibetans had a much lower prevalence of MetS as a result of possible volunteer bias.

This study revealed that the prevalence of the components of MetS varied greatly in different ethnicities. These differences may be due to genetic factors and environmental factors, which include diet and lifestyle. A randomized dietary and behavioural interventional study showed that a Tibetan diet reduced body weight and BMI in patients with CAD and MetS [28]. This might be the reason why Tibetans in our study had a low prevalence of MetS. The increased prevalence of MetS in Korean ethnicity might also be explained by diet, since it has been shown that dietary intakes of total fat and saturated fatty acids were significantly associated with MetS [29].

The association of gender with MetS is controversial [30–32]. In this study, the association of maleness with MetS was weak and became not significant in the multivariable model. In previous studies, it have been shown that advanced age and drinking status were both associated with higher risk of MetS, while the association with exercise and smoking status was equivocal [33–35]. Our finding that Chinese of Korean and Tibetan ethnicities had the highest and lowest prevalence for MetS respectively should prompt further studies to explore the possible causes of ethnic difference in the risk of developing MetS. Such studies should include a detailed dietary and lifestyle survey, and a study of socioeconomic and genetic factors.

### Limitations

There were some limitations in this study. China is a large country, so 24,796 participants represent only a small fraction of the total population. We were unable to study minorities in very remote parts of China. Different ethnic groups have different religious beliefs and customs, and these may account for some of the differences.

## Conclusions

In this large multi-ethnic population-based survey, the age-standardized prevalence of MetS varied greatly in different ethnic groups, ranging from 6·18% to 35·43%. Korean ethnicity was associated with a higher prevalence of MetS and its components, while Tibetan ethnicity was associated with a lower prevalence of MetS and its components except decreased HDL cholesterol.

## Supplementary information

**Additional file 1: Table S1.** Characteristics of included participants stratified by ethnic groups. **Table S2.** Characteristics of included participants stratified by provinces. **Table S3.** Region, ethnicity and gender specific characteristics of participants stratified by provinces**.**

## Data Availability

The datasets used and/or analysed during the current study are available on reasonable request.

## Abbreviations

BMI: Body mass index
CAD: Coronary artery disease
CVD: Cardiovascular disease
DBP: Diastolic blood pressure
GLU: Fasting blood glucose
HDL cholesterol: High-density lipoprotein cholesterol
IDF: World Health Organization (WHO) and International Diabetes Federation
IFG: Impaired fasting glucose
KNHANES: Korean National Health and Nutrition Examination Survey
LDL cholesterol: Low-density lipoprotein cholesterol
MetS: Metabolic syndrome
NCEP: ATPIII: The National Cholesterol Education Program’s Adult Treatment Panel III
ORs, 95% CI: Odds ratios and 95% confidential interval
SBP: Systolic blood pressure
T2DM: Type 2 diabetes
WC: Waist circumference
