# Supplementary material for: Prevalence of metabolic syndrome among ethnic groups in China
Source: BMC Public Health. 2020 Mar 6;20:297. doi: 10.1186/s12889-020-8393-6 (PMC7060543; doi:10.1186/s12889-020-8393-6)
Supplement: Supplementary file 1 — Additional file 1: Table S1. Characteristics of included participants stratified by ethnic groups. Table S2. Characteristics of included participants stratified by provinces. Table S3. Region, ethnicity and gender specific characteristics of participants stratified by provinces. [file 12889_2020_8393_MOESM1_ESM.docx]

**Supplementary Table 1. Characteristics of included participants stratified by ethnic groups**

| Ethnic groups | No. of participants | Age | BMI (kg/m^2^) | WC  (cm) | SBP  (mmHg) | DBP  (mmHg) | GLU (mmol/L) | TG (mmol/L) | TC (mmol/L) | HDL-C (mmol/L) | LDL-C (mmol/L) | Smoking (%) | Drinking (%) | Exercise (%) |
| --- | --- | --- | --- | --- | --- | --- | --- | --- | --- | --- | --- | --- | --- | --- |
| Han | 15867 | 25·6  (15·1,46·0) | 21·5  (19·1,24·2) | 73·0  (66·0,82·0) | 118·0  (109·0,128·0) | 75·0  (68·0,82·0) | 5·1  (4·8,5·4) | 1·0  (0·7,1·5) | 4·2  (3·6,4·9) | 1·3  (1·1,1·6) | 2·3  (1·8,2·9) | 15·4% | 17·0% | 74·8% |
| Li | 1916 | 30·0  (16·0,41·0) | 20·9  (19·1,23·0) | 70·8  (66·0,76·2) | 122·0  (112·0,133·0) | 77·0  (70·0,85·0) | 5·2  (4·9,5·4) | 1·1  (0·8,1·7) | 4·6  (3·9,5·4) | 1·5  (1·3,1·7) | 2·7  (2·2,3·3) | 17·9% | 14·1% | 32·2% |
| Miao | 453 | 26·0  (14·0,45·5) | 20·6  (17·9,23·8) | 70·0  (63·0,82·0) | 113·0  (104·0,123·0) | 73·0  (67·0,80·0) | 4·8  (4·4,5·1) | 0·9  (0·7,1·3) | 3·8  (3·3,4·4) | 1·3  (1·1,1·5) | 1·9  (1·6,2·4) | 21·1% | 16·7% | 89·0% |
| Mongolian | 1574 | 17·2  (12·4,34·6) | 20·3  (17·9,23·3) | 69·5  (62·5,78·0) | 115·0  (107·0,125·0) | 73·0  (67·0,80·0) | 4·9  (4·6,5·2) | 0·9  (0·7,1·3) | 4·2  (3·7,4·9) | 1·4  (1·2,1·6) | 2·1  (1·5,2·5) | 9·3% | 10·5% | 84·5% |
| Korean | 1223 | 17·3  (14·9,47·0) | 21·6  (19·5,24·4) | 73·0  (67·0,81·0) | 118·0  (109·0,128·0) | 75·0  (68·0,83·0) | 5·2  (4·8,5·5) | 1·1  (0·8,1·6) | 4·0  (3·5,4·7) | 1·2  (1·0,1·4) | 2·1  (1·7,2·8) | 14·4% | 17·4% | 68·7% |
| Hui | 1817 | 19·2  (14·9,38·3) | 21·1  (19·0,24·0) | 70·0  (64·0,78·0) | 119·0  (109·3,130·0) | 75·0  (68·0,83·0) | 4·9  (4·6,5·2) | 0·9  (0·6,1·3) | 3·7  (3·2,4·3) | 1·2  (1·0,1·4) | 2·1  (1·7,2·6) | 14·5% | 12·7% | 54·2% |
| Tujia | 574 | 32·0  (17·0,45·0) | 21·5  (18·7,24·5) | 73·0  (65·0,83·0) | 115·0  (106·0,124·0) | 73·0  (67·0,80·0) | 4·7  (4·3,5·0) | 1·0  (0·7,1·5) | 3·9  (3·4,4·5) | 1·2  (1·0,1·5) | 2·1  (1·6,2·6) | 26·0% | 25·1% | 87·8% |
| Tibetan | 1372 | 16·7  (14·0,31·3) | 20·5  (18·4,22·9) | 69·3  (63·4,75·5) | 111·0  (103·0,120·0) | 69·0  (63·0,76·0) | 4·9  (4·6,5·2) | 0·8  (0·6,1·1) | 3·9  (3·4,4·4) | 1·4  (1·2,1·6) | 2·0  (1·6,2·4) | 10·3% | 13·0% | 83·9% |

Abbreviations used in Table 1: BMI, body mass index; WC, waist circumference; SBP: systolic blood pressure; DBP: diastolic blood pressure; GLU: fasting blood glucose; TG: triglycerides; TC: total cholesterol; HDL-C: high density lipoprotein cholesterol; LDL-C: low density lipoprotein cholesterol.

**Supplementary Table 2. Characteristics of included participants stratified by provinces**

| Provinces | Sichuan | | Heilongjiang | | Hunnan | | | Inner Mongolia | | | Yunnan | | Ningxia | |
| --- | --- | --- | --- | --- | --- | --- | --- | --- | --- | --- | --- | --- | --- | --- |
| Ethnic groups | Han | Tibetan | Han | Korean | Han | Miao | Tujia | Han | | Mogolian | Han | Li | Han | Hui |
| No. of participants | 3134 | 1372 | 2834 | 1223 | 1823 | 453 | 574 | 3268 | | 1574 | 2822 | 1916 | 1986 | 1817 |
| Age | 36·5 (18·3,55·9) | 16·7 (14·0,31·3) | 22·4 (15·6,42·4) | 17·3 (14·9,47·0) | 31·0 (18·0,50·0) | 26·0 (14·0,45·5) | 32·0 (17·0,45·0) | 24·1 (14·2,46·0) | | 17·2  (12·4,34·6) | 19·0  (14·0,39·0) | 30·0  (16·0,41·0) | 20·1  (13·5,42·1) | 19·2  (14·9,38·3) |
| BMI (kg/m^2^) | 22·12g·6 | 21·01g·5 | 22·22g·8* | 22·12g·6* | 21·61g·3 | 21·21g·0 | 21·71g·9 | 21·51g·4 | 20·60g·6 | | 21·21g·6* | 21·21g·2* | 21·81g·0* | 21·71g·7* |
| WC (cm) | 76·26m)·5 | 70·40m)·6 | 76·26m)·1 | 74·44m)·5 | 74·84m)·0 | 72·42m)·4 | 74·44m)·0 | 73·73m)·9 | 70·70m)·9 | | 73·03m)·2 | 71·61m·7 | 71·71m)·4 | 71·41m)·2 |
| SBP (mmHg) | 124·824 ·2 | 112·912 ·7 | 117·217 ·8 | 120·420 ·3 | 116·716 ·1 | 113·713 ·1 | 115·315 ·6 | 118·618 ·5 | 116·916 ·0 | | 116·816 ·6 | 123·723 ·9 | 121·421 ·8 | 121·121 ·5 |
| DBP (mmHg) | 76·56P ·5 | 69·79P ·8 | 73·93P ·2 | 75·95P ·6 | 73·43P·5* | 73·33P·7* | 73·63P·5* | 76·56P·6 | 74·04P ·8 | | 74·44P1·1 | 77·97P ·5 | 76·56P ·0 | 76·06P ·4 |
| GLU (mmol/L) | 4·9  (4·5, 5·3) | 4·9  (4·6,5·2) | 5·2  (4·9,5·5) | 5·2  (4·8,5·5) | 5·1  (4··6,5·5) | 4·8  (4·4,5·1) | 4·7  (4·3,5·0) | 5·1  (4·8,5·4) | 4·9  (4·6,5·2) | | 5·2  (5·0,5·5) | 5·2  (4•9,5•4) | 5·0  (4·7,5·2) | 4·9  (4·6,5·2) |
| TG (mmol/L) | 1·0  (0·7, 1·5) | 0·8  (0·6,1·1) | 1·0  (0·7,1·4) | 1·1  (0·8,1·6) | 1·1  (0·7,1·5) | 0·9  (0·7,1·3) | 1·0  (0·7,1·5) | 1·1  (0·8,1·5) | 0·9  (0·7,1·3) | | 1·0  (0·7,1·5) | 1·1  (0·8,1·7) | 0·9  (0·6,1·5) | 0·9  (0·6,1·3) |
| TC (mmol/L) | 4·4C ·0 | 4·0C ·9 | 4·2C ·9 | 4·2C ·9 | 4·5C ·1 | 3·9C ·9 | 4·0C ·8 | 4·4C ·0* | 4·3* ·9* | | 4·3* ·0 | 4·7* ·1 | 3·9* ·9 | 3·8* ·9 |
| HDL-C (mmol/L) | 1·4DL·3 | 1·4DL·3 | 1·3DL·3 | 1·2DL·3 | 1·4DL·4 | 1·3DL·3 | 1·3DL·3 | 1·3DL·3 | 1·4DL·3 | | 1·5DL·3* | 1·5*L·4* | 1·2*L·3 | 1·2*L·3 |
| LDL-C (mmol/L) | 2·6DL·9 | 2·1DL·7 | 2·1DL·8 | 2·3DL·9 | 2·5DL·9 | 2·0DL·7 | 2·1DL·7 | 1·9DL·6* | | 2·0*L·6* | 2·5*L·8 | 2·8*L·9 | 2·2*L·7 | 2·2*L·7 |
| Smoking (%) | 11·5%* | 10·3%* | 15·2%* | 14·4%* | 18·5% | 21·1% | 26·0% | 15·8% | | 9·3% | 14·9% | 17·9% | 19·9% | 6·5% |
| Drinking (%) | 13·4%* | 13%* | 18·9%* | 17·4%* | 19·6% | 16·7% | 25·1% | 16·7% | | 10·5% | 13·9%* | 14·1%* | 24·0% | 12·7% |
| Exercise (%) | 81·0% | 83·9% | 79·0% | 68·7% | 75·9% | 89·0% | 87·8% | 84·2%* | | 84·5%* | 67·7% | 32·2% | 52·3%* | 54·2%* |

Abbreviations used in Supplementary Table 1: BMI: body mass index;WC: waist circumference; SBP: systolic blood pressure; DBP: diastolic blood pressure; GLU: fasting blood glucose; TG: triglycerides; TC: total cholesterol; HDL-C: high density lipoprotein cholesterol; LDL-C: low density lipoprotein cholesterol•

* No significant difference between ethnic groups•

**Suppementary Table 3 Region, ethnicity and gender specific characteristics of participants stratified by provinces**

| province | ethnic groups | gender | N | Age | BMI  (kg/m2) | WC  (cm) | SBP  (mmHg) | DBP  (mmHg) | GLU  (mmol/L) | TG  (mmol/L) | TC  (mmol/L) | HDL-C  (mmol/L) | LDL-C  (mmol/L) | Smoking  (%) | Drinking  (%) | Exercise  (%) |
| --- | --- | --- | --- | --- | --- | --- | --- | --- | --- | --- | --- | --- | --- | --- | --- | --- |
| Si Chuan | Han | M | 1491 | 27·9  (18·0,57·0) | 21·6  (19·1,24·4) | 75·0  (68·0,84·0) | 123·0  (112·0,135·0) | 76·0  (68·0,84·0) | 4·9  (4·5,5·3) | 0·9  (0·7,1·5) | 4·2  (3·6,4·9) | 1·3  (1·1,1·5) | 2·4  (1·9,3·1) | 23·4% | 25·2% | 81·1% |
|  |  | F | 1643 | 40·1  (20·4,55·5) | 22·0  (19·9,24·8) | 75·0  (69·0,83·0) | 120·0  (109·0,136·0) | 75·0  (69·0,83·0) | 4·9  (4·6,5·3) | 1·1  (0·8,1·6) | 4·5  (3·8,5·2) | 1·5  (1·3,1·7) | 2·6  (2·1,3·3) | 0·7% | 2·7% | 80·9% |
|  |  | Total | 3134 | 36·5  (18·3,55·9) | 21·9  (19·5,24·6) | 75·0  (68·3,83·5) | 121·0  (111·0,135·0) | 76·0  (69·0,83·0) | 4·9  (4·5,5·3) | 1·0  (0·7,1·5) | 4·3  (3·7,5·1) | 1·4  (1·2,1·6) | 2·5  (2·0,3·2) | 11·5%* | 13·4%* | 81·0% |
|  | Tibetan | M | 586 | 16·6  (14·2,30·3) | 19·9  (18·0,22·2) | 69·2  (63·0,76·5) | 112·0  (104·0,124·0) | 68·0  (61·0,76·0) | 4·9  (4·6,5·2) | 0·8  (0·6,1·1) | 3·7  (3·3,4·3) | 1·3  (1·1,1·5) | 1·9  (1·5,2·4) | 23·3% | 26·5% | 84·6% |
|  |  | F | 786 | 16·9  (13·7,32·4) | 20·9  (18·7,23·3) | 69·5  (63·8,75·3) | 110·0  (102·0,118·0) | 70·0  (64·0,77·0) | 4·8  (4·6,5·1) | 0·8  (0·6,1·2) | 4·0  (3·5,4·5) | 1·4  (1·2,1·6) | 2·0  (1·7,2·5) | 0·9% | 3·1% | 83·3% |
|  |  | Total | 1372 | 16·7  (14·0,31·3) | 20·5  (18·4,22·9) | 69·3  (63·4,75·5) | 111·0  (103·0,120·0) | 69·0  (63·0,76·0) | 4·9  (4·6,5·2) | 0·8  (0·6,1·1) | 3·9  (3·4,4·4) | 1·4  (1·2,1·6) | 2·0  (1·6,2·4) | 10·3%* | 13%* | 83·9% |
| Hei Longjiang | Han | M | 1356 | 21·6  (15·4,40·0) | 22·5  (19·6,25·7) | 79·0  (70·0,89·0) | 120·0  (112·0,129·0) | 74·0  (67·0,81·0) | 5·3  (4·9,5·6) | 1·1  (0·7,1·6) | 4·0  (3·5,4·8) | 1·2  (1·0,1·4) | 2·1  (1·5,2·7) | 30·9% | 35·1% | 76·5% |
|  |  | F | 1478 | 23·8  (15·7,44·3) | 21·4  (19·2,23·9) | 72·0  (66·0,79·0) | 113·0  (105·8,121·0) | 73·0  (67·0,80·0) | 5·1  (4·9,5·4) | 0·9  (0·7,1·3) | 4·2  (3·6,4·8) | 1·3  (1·1,1·5) | 2·0  (1·6,2·5) | 0·8% | 4·4% | 81·3% |
|  |  | Total | 2834 | 22·4  (15·6,42·4) | 21·8  (19·4,24·9)* | 75·0  (68·0,84·0) | 117·0  (108·0,125·0) | 73·0  (67·0,80·0) | 5·2  (4·9,5·5) | 1·0  (0·7,1·4) | 4·1  (3·5,4·8) | 1·3  (1·1,1·5) | 2·0  (1·6,2·6) | 15·2%* | 18·9%* | 79·0% |
|  | Korean | M | 516 | 18·2  (15·0,50·1) | 21·9  (19·4,24·9) | 77·0  (69·0,85·0) | 122·0  (114·0,132·0) | 76·0  (69·0,85·0) | 5·3  (4·8,5·6) | 1·1  (0·8,1·8) | 3·9  (3·4,4·6) | 1·1  (1·0,1·3) | 2·1  (1·6,2·8) | 33·7% | 34·5% | 66·1% |
|  |  | F | 707 | 16·8  (14·9,45·5) | 21·5  (19·5,24·1) | 71·0  (66·0,78·0) | 116·0  (107·0,126·0) | 74·0  (67·0,81·0) | 5·1  (4·7,5·5) | 1·1  (0·8,1·5) | 4·1  (3·6,4·7) | 1·2  (1·0,1·4) | 2·2  (1·7,2·8) | 0·6% | 5·3% | 70·6% |
|  |  | Total | 1223 | 17·3  (14·9,47·0) | 21·6  (19·5,24·4)* | 73·0  (67·0,81·0) | 118·0  (109·0,128·0) | 75·0  (68·0,83·0) | 5·2  (4·8,5·5) | 1·1  (0·8,1·6) | 4·0  (3·5,4·7) | 1·2  (1·0,1·4) | 2·1  (1·7,2·8) | 14·4%* | 17·4%* | 68·7% |
| Hu Nan | Han | M | 930 | 34·0  (19·0,53·0) | 22·4  (19·5,24·8) | 79·0  (69·0,87·0) | 120·0  (112·0,129·0) | 75·0  (68·3,82·0) | 5·2  (4·8,5·7) | 1·2  (0·8,1·7) | 4·4  (3·7,5·2) | 1·3  (1·1,1·5) | 2·4  (1·9,3·2) | 35·1% | 35·4% | 75·5% |
|  |  | F | 893 | 28·0  (18·0,46·0) | 20·7  (19·1,22·7) | 70·0  (65·0,76·0) | 111·0  (103·0,120·0) | 71·0  (65·8,77·0) | 5·0  (4·6,5·4) | 1·0  (0·7,1·3) | 4·3  (3·8,5·0) | 1·5  (1·2,1·7) | 2·3  (1·8,2·9) | 1·5% | 3·3% | 76·4% |
|  |  | Total | 1823 | 31·0  (18·0,50·0) | 21·3  (19·3,23·9) | 73·0  (66·0,83·0) | 116·0  (107·0,125·0) | 73·0  (67·0,79·0)* | 5·1  (4·6,5·5) | 1·1  (0·7,1·5) | 4·4  (3·7,5·1) | 1·3  (1·1,1·6) | 2·4  (1·8,3·0) | 18·5% | 19·6% | 75·9% |
|  | Miao | M | 258 | 32·0  (14·0,46·0) | 20·9  (18·1,24·8) | 73·0  (65·0,87·0) | 116·0  (106·0,125·0) | 74·0  (67·0,81·0) | 4·8  (4·4,5·1) | 0·9  (0·7,1·4) | 3·8  (3·3,4·5) | 1·2  (1·0,1·5) | 1·9  (1·6,2·5) | 36·8% | 28·9% | 89·5% |
|  |  | F | 195 | 17·0  (14·0,44·0) | 20·2  (17·4,23·1) | 67·5  (60·8,75·0) | 109·0  (102·0,118·0) | 72·0  (66·0,79·0) | 4·6  (4·3,5·0) | 0·9  (0·6,1·2) | 3·7  (3·3,4·2) | 1·3  (1·1,1·5) | 1·9  (1·6,2·3) | 0·0% | 0·5% | 88·2% |
|  |  | Total | 453 | 26·0  (14·0,45·5) | 20·6  (17·9,23·8) | 70·0  (63·0,82·0) | 113·0  (104·0,123·0) | 73·0  (67·0,80·0)* | 4·8  (4·4,5·1) | 0·9  (0·7,1·3) | 3·8  (3·3,4·4) | 1·3  (1·1,1·5) | 1·9  (1·6,2·4) | 21·1% | 16·7% | 89·0% |
|  | Tujia | M | 335 | 33·0  (17·0,47·0) | 22·2  (19·1,25·4) | 78·0  (69·0,87·0) | 118·0  (109·0,128·0) | 75·0  (68·0,82·0) | 4·7  (4·4,5·1) | 1·1  (0·8,1·7) | 4·0  (3·4,4·6) | 1·2  (1·0,1·4) | 2·2  (1·7,2·6) | 44·6% | 41·6% | 88·7% |
|  |  | F | 239 | 31·0  (15·0,43·0) | 20·5  (18·5,22·9) | 68·0  (63·0,75·3) | 110·0  (102·8,118·0) | 71·0  (65·0,78·0) | 4·6  (4·3,5·0) | 0·9  (0·6,1·2) | 3·7  (3·3,4·4) | 1·3  (1·1,1·6) | 2·0  (1·6,2·5) | 0·0% | 2·1% | 86·6% |
|  |  | Total | 574 | 32·0  (17·0,45·0) | 21·5  (18·7,24·5) | 73·0  (65·0,83·0) | 115·0  (106·0,124·0) | 73·0  (67·0,80·0)* | 4·7  (4·3,5·0) | 1·0  (0·7,1·5) | 3·9  (3·4,4·5) | 1·2  (1·0,1·5) | 2·1  (1·6,2·6) | 26·0% | 25·1% | 87·8% |
| Inner Mogolia | Han | M | 1500 | 23·6  (14·0,47·6) | 21·7  (18·5,24·5) | 76·0  (66·0,86·0) | 120·0  (112·0,129·0) | 77·0  (70·0,83·0) | 5·2  (4·9,5·5) | 1·1  (0·8,1·6) | 4·2  (3·6,5·1) | 1·3  (1·1,1·5) | 2·0  (1·6,2·4) | 31·2% | 32·0% | 83·1% |
|  |  | F | 1768 | 24·6  (14·5,45·2) | 21·5  (19·0,24·0) | 71·0  (65·0,78·0) | 115·0  (108·0,123·0) | 76·0  (70·0,81·0) | 5·1  (4·8,5·4) | 1·1  (0·9,1·5) | 4·3  (3·7,5·0) | 1·4  (1·2,1·6) | 1·8  (1·5,2·2) | 2·6% | 3·6% | 85·1% |
|  |  | Total | 3268 | 24·1  (14·2,46·0) | 21·6  (18·8,24·2)* | 73·0  (65·0,82·0) | 118·0  (110·0,126·0) | 76·0  (70·0,82·0) | 5·1  (4·8,5·4) | 1·1  (0·8,1·5) | 4·3  (3·6,5·1)* | 1·3  (1·2,1·5) | 1·9  (1·5,2·3)* | 15·8% | 16·7% | 84·2%* |
|  | Mogolian | M | 661 | 15·6  (12·0,30·2) | 19·9  (17·4,23·4) | 70·0  (62·0,81·0) | 118·0  (110·0,127·0) | 74·0  (67·0,81·5) | 4·9  (4·7,5·2) | 1·0  (0·7,1·4) | 4·3  (3·7,4·9) | 1·4  (1·2,1·6) | 2·3  (1·5,2·6) | 19·9% | 21·4% | 83·5% |
|  |  | F | 913 | 19·3  (13·2,37·5) | 20·5  (18·2,23·3) | 69·0  (63·0,76·0) | 114·0  (106·0,122·0) | 73·0  (67·0,80·0) | 4·8  (4·6,5·1) | 0·9  (0·7,1·3) | 4·2  (3·7,4·8) | 1·4  (1·2,1·6) | 2·0  (1·4,2·2) | 1·7% | 2·7% | 85·2% |
|  |  | Total | 1574 | 17·2  (12·4,34·6) | 20·3  (17·9,23·3)* | 69·5  (62·5,78·0) | 115·0  (107·0,125·0) | 73·0  (67·0,80·0) | 4·9  (4·6,5·2) | 0·9  (0·7,1·3) | 4·2  (3·7,4·9)* | 1·4  (1·2,1·6) | 2·1  (1·5,2·5)* | 9·3% | 10·5% | 84·5%* |
| Yun Nan | Han | M | 1417 | 20·0  (14·0,38·0) | 20·9  (18·6,23·8) | 73·0  (67·0,82·0) | 118·0  (110·0,128·0) | 74·0  (67·0,82·0) | 5·3  (5·0,5·6) | 1·0  (0·7,1·5) | 4·2  (3·6,4·9) | 1·4  (1·2,1·6) | 2·4  (1·9,3·0) | 29·2% | 25·1% | 67·0% |
|  |  | F | 1405 | 18·0  (14·0,39·0) | 20·8  (18·8,23·0) | 70·2  (65·0,77·0) | 113·0  (105·0,123·0) | 74·0  (67·0,81·0) | 5·2  (4·9,5·5) | 1·0  (0·7,1·4) | 4·3  (3·7,4·9) | 1·5  (1·3,1·7) | 2·4  (1·9,2·9) | 0·5% | 2·5% | 68·3% |
|  |  | Total | 2822 | 19·0  (14·0,39·0) | 20·8  (18·7,23·4)* | 72·0  (66·0,79·2) | 116·0  (107·0,126·0) | 74·0  (67·0,81·0) | 5·2  (5·0,5·5) | 1·0  (0·7,1·5) | 4·2  (3·7,4·9) | 1·5  (1·3,1·7)* | 2·4  (1·9,3·0) | 14·9% | 13·9%* | 67·7% |
|  | Li | M | 963 | 28·0  (15·0,40·0) | 20·8  (18·8,23·1) | 71·0  (66·2,77·0) | 123·0  (114·0,134·0) | 77·0  (70·0,85·0) | 5·2  (4·9,5·5) | 1·1  (0·8,1·7) | 4·6  (3·9,5·4) | 1·5  (1·3,1·7) | 2·7  (2·2,3·4) | 28·4% | 23·1% | 37·0% |
|  |  | F | 953 | 32·0  (16·0,42·0) | 20·9  (19·3,23·0) | 70·2  (65·4,76·0) | 120·5  (110·8,133·0) | 78·0  (71·0,85·0) | 5·1  (4·8,5·4) | 1·1  (0·8,1·7) | 4·6  (4·0,5·3) | 1·5  (1·3,1·7) | 2·7  (2·2,3·3) | 7·1% | 5·0% | 27·3% |
|  |  | Total | 1916 | 30·0  (16·0,41·0) | 20·9  (19·1,23·0)^*^ | 70·8  (66·0,76·2) | 122·0  (112·0,133·0) | 77·0  (70·0,85·0) | 5·2  (4·9,5·4) | 1·1  (0·8,1·7) | 4·6  (3·9,5·4) | 1·5  (1·3,1·7)^*^ | 2·7  (2·2,3·3) | 17·9% | 14·1%^*^ | 32·2% |
| Ning Xia | Han | M | 1015 | 20·1  (13·8,43·8) | 21·7  (18·8,25·2) | 73·0  (64·6,83·0) | 122·0  (111·0,134·0) | 76·0  (68·0,86·0) | 5·0  (4·7,5·3) | 1·0  (0·6,1·5) | 3·7  (3·2,4·3) | 1·1  (1·0,1·3) | 2·1  (1·6,2·6) | 36·9% | 41·4% | 51·5% |
|  |  | F | 971 | 20·1  (13·4,40·4) | 21·4  (18·9,23·7) | 68·0  (62·0,75·5) | 116·0  (107·0,127·0) | 76·0  (69·0,83·0) | 5·0  (4·7,5·2) | 0·9  (0·6,1·4) | 3·8  (3·3,4·5) | 1·2  (1·1,1·4) | 2·2  (1·8,2·7) | 1·2% | 4·9% | 53·1% |
|  |  | Total | 1986 | 20·1  (13·5,42·1) | 21·5  (18·8,24·4)^*^ | 70·0  (63·0,80·0) | 119·0  (109·0,130·0) | 76·0  (68·0,84·0) | 5·0  (4·7,5·2) | 0·9  (0·6,1·5) | 3·8  (3·2,4·4) | 1·2  (1·0,1·4) | 2·2  (1·7,2·7) | 19·9% | 24·0% | 52·3%* |
|  | Hui | M | 890 | 20·0  (15·2,41·9) | 21·2  (19·0,24·8) | 72·0  (66·0,82·7) | 122·0  (112·0,133·0) | 76·0  (68·0,85·0) | 4·9  (4·7,5·2) | 0·9  (0·7,1·5) | 3·6  (3·1,4·2) | 1·1  (0·9,1·2) | 2·1  (1·6,2·6) | 28·0% | 21·1% | 53·7% |
|  |  | F | 927 | 18·8  (14·7,34·5) | 21·0  (19·0,23·5) | 68·0  (63·0,73·0) | 116·0  (108·0,126·0) | 75·0  (68·0,82·0) | 4·9  (4·6,5·2) | 0·9  (0·6,1·3) | 3·7  (3·3,4·3) | 1·3  (1·1,1·4) | 2·1  (1·7,2·6) | 0·7% | 4·3% | 54·6% |
|  |  | Total | 1817 | 19·2  (14·9,38·3) | 21·1  (19·0,24·0)^*^ | 70·0  (64·0,78·0) | 119·0  (109·3,130·0) | 75·0  (68·0,83·0) | 4·9  (4·6,5·2) | 0·9  (0·6,1·3) | 3·7  (3·2,4·3) | 1·2  (1·0,1·4) | 2·1  (1·7,2·6) | 6·5% | 12·7% | 54·2%* |

Abbreviations used in Supplementary Table 2: BMI: body mass index;WC: waist circumference; SBP: systolic blood pressure; DBP: diastolic blood pressure; GLU: fasting blood glucose; TG: triglycerides; TC: total cholesterol; HDL-C: high density lipoprotein cholesterol; LDL-C: low density lipoprotein cholesterol.

* No significant difference between ethnic groups.
